# Mesenchymal stromal cells as carriers of IL-12 reduce primary and metastatic tumors of murine melanoma

**DOI:** 10.1038/s41598-021-97435-9

**Published:** 2021-09-15

**Authors:** Natalia Kułach, Ewelina Pilny, Tomasz Cichoń, Justyna Czapla, Magdalena Jarosz-Biej, Marek Rusin, Alina Drzyzga, Sybilla Matuszczak, Stanisław Szala, Ryszard Smolarczyk

**Affiliations:** Center for Translational Research and Molecular Biology of Cancer, Maria Skłodowska-Curie National Research Institute of Oncology, Gliwice Branch, Wybrzeże Armii Krajowej Street 15, 44-102 Gliwice, Poland

**Keywords:** Cancer, Cancer microenvironment, Cancer therapy, Skin cancer, Biotechnology, Gene therapy

## Abstract

Due to immunosuppressive properties and confirmed tropism towards cancer cells mesenchymal stromal cells (MSC) have been used in many trials. In our study we used these cells as carriers of IL-12 in the treatment of mice with primary and metastatic B16-F10 melanomas. IL-12 has confirmed anti-cancer activity, induces a strong immune response against cancer cells and acts as an anti-angiogenic agent. A major limitation of the use of IL-12 in therapy is its systemic toxicity. The aim of the work was to develop a system in which cytokine may be administered intravenously without toxic side effects. In this study MSC were used as carriers of the IL-12. We confirmed antitumor effectiveness of the cells secreting IL-12 (MSC/IL-12) in primary and metastatic murine melanoma models. We observed inhibition of tumor growth and a significant reduction in the number of metastases in mice after MSC/IL-12 administration. MSC/IL-12 decreased vascular density and increased the number of anticancer M1 macrophages and CD8^+^ cytotoxic T lymphocytes in tumors of treated mice. To summarize, we showed that MSC are an effective, safe carrier of IL-12 cytokine. Administered systemically they exert therapeutic properties of IL-12 cytokine without toxicity. Therapeutic effect may be a result of pleiotropic (proinflammatory and anti-angiogenic) properties of IL-12 released by modified MSC.

## Introduction

The results of in vitro studies on molecular anticancer agents are promising. However, the efficacy of therapeutic factors in vivo is limited. It is due to the presence of anatomical and physiological barriers in the structure and functioning of tumors, their heterogeneity, invasiveness and the immune system activity. Therapeutic factors often degrade before they reach their destination or exert systemic toxicity. The research into the precise delivery of therapeutic agents to cancer is an important element in design of new anti-cancer strategies^1^.

### The “Trojan horse” strategy in cell therapy

For years, the idea of using cells as carriers of therapeutic agents has attracted the attention of researchers. The Endothelial Precursor Cells (EPC), which were able to migrate specifically to the place of the formation and reconstruction of blood vessels, were already studied in the 90's. Attempts were also made to use macrophages^2^ and neutrophils^1^ as carriers of therapeutic factors. Transporting cells were armed, among others, with cytotoxic proteins (e.g., TRAIL), immunomodulatory agents, antibodies (e.g., anti-HER2, human epidermal growth factor receptor 2), oncolytic viruses (e.g., Ad5-survivin-pk7), nanoparticles^3^.

### MSC as carriers of anticancer agent

MSC have the characteristics of an ideal carrier. They are easily accessible cells showing strong tropism to inflamed tissues and tumors. Immunologically privileged—they do not evoke immune response after transplantation, exert immunomodulatory properties in inflammatory environment and do not provoke an attack of the immune cells^4^. MSC were used as carriers of anti-cancer factors. Modified MSC expressing Herpes Simplex Virus-Thymidine Kinase (HSV-TK) localized in the vicinity of tumors. VEGF receptor producing were used in an experimental model of mice with lung and colorectal cancer. The protein secreted near the tumor cells showed an anti-angiogenic and pro-apoptotic effect^5^. MSC were used as carriers of IFN^6–9^, TNF, IL-15, IL-18, IL-21^10^, CX3CL1^11^, oncolytic viruses^12–14^, photothermal agents^15,16^, drugs (paclitaxel)^17^.

### Therapeutic agent: cytokine IL-12

In recent years, it has become clear that anti-cancer therapies targeting cancer cells alone have limited effectiveness. Cancer functions like a separate, pathologically altered tissue made of cancer cells and their microenvironment. The tumor microenvironment consists of endothelial cells, fibroblasts, immune cells, signalling molecules and extracellular matrix.

The microenvironment participates in the progression and expansion of cancer; it is responsible for the resistance to the applied therapeutic agents^18–21^.

The pleiotropic (anti-angiogenic and immunostimulatory) effect of cytokine IL-12 may contribute to a change in the nature of the tumor microenvironment (polarization) from pro-angiogenic and immunosuppressive towards anti-angiogenic and immunostimulatory and became the basis of novel anticancer therapeutic strategy.

In this study, mesenchymal stromal cells were used as carriers of the anti-tumor factor IL-12 in the therapy of mice with primary and metastatic melanomas.

## Materials and methods

### Cells lines and animals

B16-F10 (murine melanoma) cell line (ATCC, Manassas, VA, USA) used to obtain primary tumors and metastases of murine melanoma was maintained using RPMI 1640 medium (Gibco BRL, Paisley, UK) supplemented with 10% fetal bovine serum (Thermo Fisher Scientific, USA). GL261 (mouse glioma) cell line (PerkinElmer, Waltham, MA, USA) was maintained using DMEM (Gibco BRL, Paisley, UK) supplemented with 10% fetal bovine serum (Thermo Fisher Scientific, USA). Cells cultures were kept under standard conditions (37 °C, 5% CO_2_, 95% humidity).

Experiments on animals were carried out in accordance with standard procedures. The study was approved by the Local Ethics Commission at the Medical University of Silesia in Katowice (approval no. 54/2018). The study was carried out in compliance with the ARRIVE guidelines. Mice (6 to 8-weeks-old, C57Bl/6NCrl) were obtained from Charles River Breeding Laboratories. All mice were housed in the Maria Sklodowska-Curie National Research Institute of Oncology, Gliwice Branch (Poland) in a HEPA-filtered Allentown’s IVC System (Allentown Caging Equipment Co, NJ, USA). The animals received a total pathogen-free standard diet (Altromin 1314, Altromin Spezialfutter GmbH & Co. KG, Germany) and water ad libitum throughout the whole study. The animals were monitored every day. This study was carried out in accordance with the recommendations in the Guide for the Care and Use of Laboratory Animals of the National Institutes of Health. All experiments on animals were conducted in accordance with the 3R rule.

### Mesenchymal stromal cells: isolation and phenotypic characterization of mesenchymal stromal cells

The mice (6–8-week-old) were sacrificed by cervical dislocation. Femoral bones were excised and soft tissues were removed.

The bones were cut, washed with saline and incubated with collagenase solution (3 h, 37 °C, 0.4 U/ml, Serva Electrophoresis, Heidelberg, Germany). The cells suspension was filtered through 70 μm strainer. The cells were maintained in IMDM (Sigma Aldrich, St Louis, MO, USA) supplemented with 10% fetal bovine serum (Thermo Fisher Scientific), antibiotics (penicillin and streptomycin, Sigma Aldrich, USA) and murine bFGF (1 ng/ml). After 72 h, the plates were rinsed with PBS and fresh culture medium was provided. The medium was changed every 2–3 days. Cell cultures were kept under standard conditions (37 °C, 5% CO_2_, 95% humidity). The cells were cryopreserved in FBS, DMSO and IMDM solution in − 80 °C until needed.

The phenotype of the cells adherent to plastic dishes was determined using a flow cytometer (FACS Canto BD and BD FACSAria III; BD, Franklin Lakes, NJ, USA). To obtain a single cell suspension the cells were treated with 0.25% trypsin (Gibco BRL, Paisley, UK). The cells were incubated with antibodies directed against the following murine antigens: CD44, CD105, Sca-1, CD29, CD90.1, CD45, CD11b, CD106 or isotype-matched control antibodies according to the manufacturer's protocol (30 min., 4 °C, BioLegend, San Diego, CA, USA). 7-AAD (7-amino-actinomycin D) was used to stain nonviable cells (5 μl/10^6^ cells; BioLegend, San Diego, CA, USA). The cells with phenotype 7-AAD^-^CD44^+^CD105^+^Sca-1^+^CD29^+^CD90.1^+^CD45^-^CD11b^-^CD106^-^ were considered as MSC.

The ability of obtained cells to differentiate into adipocytes, chondrocytes and osteoblasts was assessed using Mouse Mesenchymal Stem Cell Functional Identification Kit (R&D, Minneapolis, MN, USA). The procedure was performed in accordance with the manufacturer’s instructions. The differentiation of MSC into chondrocytes and osteocytes was visualized by histochemical staining using Safranin O and Alizarin Red (Sigma Aldrich, USA) and observed with Eclipse 80i microscope (Nikon Instruments Inc., Melville, NY, USA). The differentiation of MSC into adipocytes was assessed by immunofluorescence staining using an antibody directed against the FABP4 (Abcam; Ab205332, 1:100, Cambridge, UK). The sections were incubated with secondary antibodies conjugated with fluorochromes (FITC) (Vector Laboratories, FI-1200, 1:100; Burlingame, USA). Fluorescence imaging of the stained cells was performed using a LSM710 confocal microscope (Carl Zeiss Microscopy GmbH, Germany).

### MSC tropism towards cancer cells, in vitro examination in Boyden Chambers

Tropism towards tumor cells was examined in vitro using Boyden chambers (Corning Life Sciences, NY, USA). MSC or MSC/IL-12 were placed on a cylindrical cell culture insert with porous bottom nested inside the well of a cell culture plate filled with tested medium. The tested media were collected from over the B16-F10 and GL261 cell cultures, as control media fresh IMDM media with and without FBS supplementation were used. The cells that migrated through the pores of the cellulose membrane were fixed in cold methanol and stained with Giemsa solution (Merck Milipore, Darmstadt, Germany). The cells were counted in 5 fields of view of Eclipse 80i microscope at × 10 magnification.

### MSC tropism towards glioma cells in Matrigel

Co-culture of MSC and GL261eGFP cells in Matrigel medium. MSC were stained with the PKH26 Red Fluorescent Cell Linker Kit (Sigma Aldrich, USA) according to the manufacturer's protocol. Single cells suspensions of GL261eGFP and stained MSC were mixed in equal numbers, suspended in IMDM (3 × 10^3^ cells/100 µl medium/well) and placed in Matrigel coated 96-well plate (according to manufacturer’s protocol). The plate was placed in the Zeiss Cell Observer SD chamber and incubated for 24 h under standard culture conditions (37 °C, 95% CO_2_). Observations were recorded using a microscope camera. In the first 5 h of the experiment, photos were taken every 30 min and then every 1 h.

### Cloning of cDNA encoding IL-12 in adenoviral vectors

pBCMGSNeo plasmid obtained from Dr. H. Yamamoto (Osaka University, Osaka, Japan) contains cDNA encoding the two subunits of IL-12. The cDNA was isolated and amplified with PCR reaction. The starters were designed based on IL-12 cDNA template with 15 bp extensions homologous to the ends of linearized adenoviral vector pAdenoX-DsRed-Express (Clontech, CA, USA). PCR product was purified and introduced into the adenoviral vector with In-Fusion cloning system (Clontech, CA, USA). The procedure was performed in accordance with the manufacturer’s instructions. Following the reaction a portion of In-Fusion mixture was transformed into *E. coli* in SOC medium (Stellar Component Cells, Clontech, CA, USA). The transformation mix was spread onto LB agar plates with ampicillin (100 μg/ml). Well-separated colonies arisen on the plates were subjected to PCR Colony Screening using Terra PCR Kit (Clontech, CA, USA). Positive clones were amplified and the plasmid was purified. Cloning correctness was confirmed by sequencing of DNA fragment incorporated into the adenoviral vector. The sequencing procedure was performed using 3500 HITACHI Genetic Analizer (Applayd Biosystem, Foster City, CA, USA). Obtained vector was linearized using PacI restriction enzyme according to manufacturer’s instructions (NewEngland Biolabs, UK).

### Preparation of the viruses

Transfection of packaging AdenoX 293 cells (Clontech, USA) with obtained vector was conducted using X-tremeGENE HP DNA Transfection Reagent (Roche, Basel, Switzerland) according to manufacturer’s instructions.

Cell lysis was performed by rapid freezing and thawing the suspension in an alcohol bath (dry ice with 80% alcohol) and in a water bath (37 °C). For the amplification of the adenoviruses, AdenoX 293 cells were transduced with obtained virus particles on 75 cm^3^ bottles according to manufacturer’s instructions.

The procedure was repeated. Viruses isolated from the 7th amplification cycle were used for the experiment. The AdenoX GoStix system (Clontech, USA) was used to confirm the presence and concentration of viral particles in the supernatant medium according to the manufacturer instructions. The aliquots obtained were stored at -20 °C until needed.

### Preparation of modified MSC

7.5 × 10^5^ MSC suspended in IMDM culture medium with FBS was placed on a 6 cm plate and incubated under standard culture conditions (37 °C, 5% CO_2_). When the cells reached 70% confluence, the medium was replaced with 1.5 ml fresh IMDM, 200 µl of the adenovirus suspension obtained in the previous steps of the procedure and polybrene (8 µg/ml). Cells were incubated (4 h, 37 °C, 5% CO_2_) then 2 ml fresh IMDM culture medium with FBS was added and incubated until intense fluorescence (24–48 h). Cells fluorescence was observed under a microscope Zeiss Cell Observer. An ELISA assay (Platinum ELISA, eBioscience, USA) was used to confirm if the modified cells produce IL-12 protein. The procedure was carried out according to the manufacturer's protocol.

#### Phenotypic characterization of modified MSC

Sterile cover glasses were placed on a 6 cm plate and 7.5 × 10^5^ modified MSC suspended in IMDM culture medium with FBS were incubated under standard culture conditions (37 °C, 5% CO_2_) for 24 h. Cover glasses were fixed with 4% paraformaldehyde, permeabilized with 0.1% Triton X-100 and stained with antibodies conjugated with fluorochrome: CD29-APC, CD90-FITC, Sca-1-APC, CD45-APC (BioLegend, USA). Microscopic observations were performed using an LSM 710 Zeiss confocal microscope (Carl Zeiss Microscopy GmGB, Gottingen, Germany).

### Treatment of mice bearing primary melanoma B16-F10 tumors

6–8 weeks mice were used for the experiment. The mice were divided into 3 experimental groups: (1) PBS, (2) MSC, (3) MSC/IL-12. Nine days after inoculating the mice (lower flank) with B16-F10 melanoma cells (2 × 10^5^ cells/100 μl PBS/mouse) MSC, MSC/IL-12 cells and PBS were administered intratumorally (5 × 10^5^ cells/100 μl PBS/mouse). Growing tumors were measured with calipers, and tumor volumes were determined using the formula: volume = width^2^ × length × 0.52.

### Treatment of mice with experimental lung metastasis of B16-F10 melanoma

6–8 weeks mice were used for the experiment. To obtain metastases in mice B16-F10 cells (2 × 10^5^ cells/100 μl PBS/mouse) were administered to the tail vein. 3 experimental groups were formed: (1) PBS, (2) MSC, (3) MSC/IL-12. On day 5th after administration of the cancer cells the mice were given MSC, MSC/IL-12 cells (5 × 10^5^ cells /100 μl PBS/mouse) and PBS to the tail vein. On day 21st the mice were sacrificed, the lungs collected and fixed in Bouin's solution. After 24 h the lungs were weighed and metastases were counted.

### Post-therapeutic analyses

#### Tumor vascularization analysis

On days 3 and 8 after MSC/IL-12 cells administrations tumors were excised, embedded in liquid nitrogen and sectioned into 5 µm slices. To determine the presence of the blood vessels in collected tumors, frozen sections were stained using antibody directed against CD31 antigen (Abcam; Ab7388, 1:50, Cambridge, UK). The sections were incubated with secondary antibodies conjugated with fluorochromes (AlexaFluor 594) (Abcam; ab150168, 1:100, Cambridge, UK) and cover slipped with Vectashield mounting medium containing DAPI (Vector Laboratories, USA). Area occupied by blood vessels was counted with ImageJ software (NIH). Stained blood vessels were counted in 5 randomly chosen fields (magn. ×20) per section in 5 tumors of each group. Microscopic observations were performed using an LSM 710 Zeiss confocal microscope (Carl Zeiss Microscopy GmGB, Gottingen, Germany).

#### Determination of the M1 and M2 macrophages presence in B16-F10 melanoma tumors after the therapy

##### Flow cytometry

On day 8 after MSC/IL-12 cells administration tumors were excised, single-cell suspension was obtained using collagenase II solution (500 U/ml; Gibco BRL, Paisley, UK). Red blood cells were lysed using 0.15 M ammonium chloride (Sigma Aldrich, St Louis, MO, USA). Dead cells were removed by centrifugation using Histopaque-1077 gradient (Sigma Aldrich, USA). To identify the subpopulations of macrophages, antibodies against the following antigens were used: CD45, F4/80, CD206 and CD86 (eBioscience, USA) or isotype-matched control antibodies. All FACS-analyzed (BD FACSCanto, BD, USA) populations were gated in a 7-AAD window to enrich for viable cells. 7-AAD^-^CD45^+^ F4/80^+^ CD86^+^ cells were considered as M1 macrophages, 7-AAD^-^CD45^+^ F4/80^+^ CD206^+^ cells were considered as M2 macrophages.

##### Immunohistochemistry

On day 8 after intratumoral MSC/IL-12 cells administration the tumors were collected, embedded in liquid nitrogen and sectioned. Frozen sections were stained using antibodies directed against CD206 antigen (1:100, Abcam, Cambridge, UK) and F4/80 antigen (1:100, Abcam; Cambridge, UK). The sections were incubated with the secondary antibodies conjugated with fluorochromes (FITC, AlexaFluor 594) (Abcam, UK; ab150168, 1:100, Vector Laboratories, Burlingame, USA UK UK). Sections were mounted in VECTASHIELD Mounting Medium with DAPI (Vector Laboratories, USA). The fluorescence intensity was measured with ImageJ software (NIH). Microscopic observations were performed using an LSM 710 Zeiss confocal microscope (Carl Zeiss Microscopy GmGB, Gottingen, Germany).

### Determination of CD8^+^ T lymphocytes in B16-F10 melanoma tumors

On day 3 after intratumoral MSC/IL-12 cells administration the tumors were collected, embedded in liquid nitrogen and sectioned. Frozen sections were stained using antibody directed against CD8^+^ antigen (1:50, Abcam, Cambridge, UK). The sections were incubated with the secondary antibody conjugated with fluorochromes (Alexa Fluor 594) (1:100, Vector Laboratories, Burlingame, USA). Sections were mounted in VECTASHIELD Mounting Medium with DAPI (Vector Laboratories, USA). The number of the of CD8^+^ T lymphocytes in each group was determined in 5 randomly chosen fields (magn. 20 ×) per section in 5 tumors of each group and calculated per 1 mm^2^ of the tumor. Microscopic observations were performed using an LSM 710 Zeiss confocal microscope (Carl Zeiss Microscopy GmGB, Gottingen, Germany).

### Statistics

Statistical analyses were performed using Statistica software (Dell Inc. (2016), version 13. software.dell.com.). The Shapiro–Wilk test was used to verify the normality of the distribution. The statistical significance of differences between the experimental and control groups were evaluated with Kruskal–Wallis and multiple comparisons of mean ranks for all groups tests using Statistica software. Differences in p values of 0.05 or less were considered significant.

## Results

### The cells

#### Mesenchymal stromal cells were isolated from murine bone marrow

**Figure 1:**
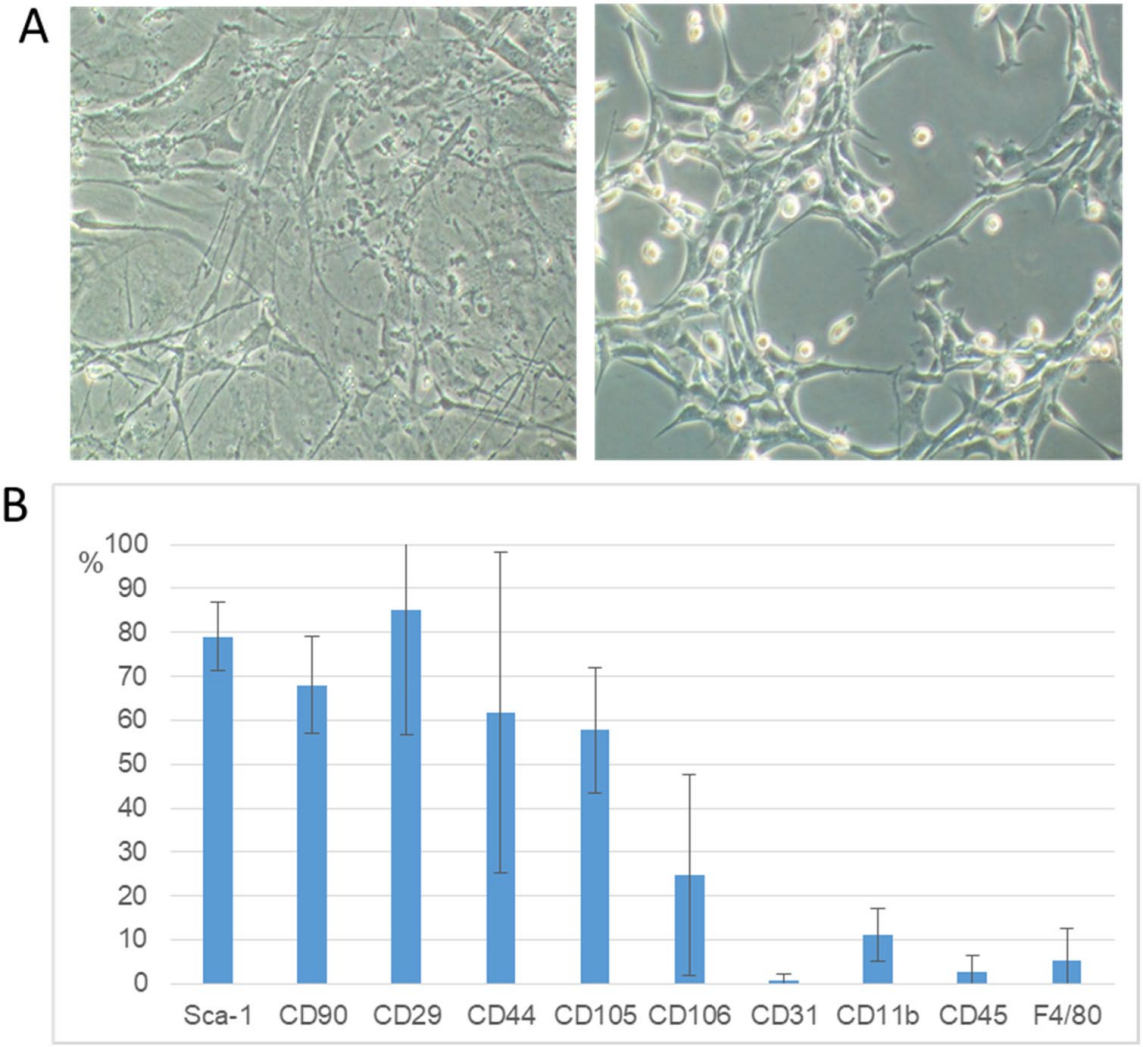
The morphology (**A**) and phenotype (**B**) of the isolated cells. Microscopic observations of the cell culture (lens magn. 5 × left, 10 × right) (**A**). Antigen profile of the isolated cells: presence of Sca-1, CD29 (approx. 80%), CD90 (approx. 70%), CD44, CD105 (approx. 60%) antigens, a small percentage of CD106 (approx. 20%) and CD11b (10%), absence of F4/80 (approx. 4%) and CD45, CD31 antigens (below 3%), (n = 5) (**B**).

The cells isolation protocol was optimized. The obtained cells met the MSC-specific criteria. The cells were grown on plastic dishes, they had a slender, fusiform shape and a fibroblast-like morphology (Fig. 1A). Using a flow cytometer, their phenotype was examined. Isolated cells had a characteristic MSC phenotype; 80% of the cells expressed Sca-1 and CD29 antigens, 70% CD90 antigen, 60% CD44 and CD105 antigens. Cells had less than 3% CD45 hematopoietic antigen and did not have endothelial CD31 antigen (less than 1%) (Fig. 1B).

**Figure 2:**
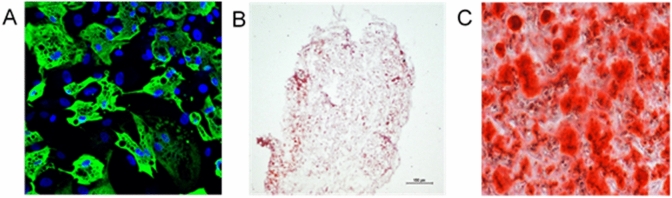
Differentiation of isolated cells. (**A**) MSC differentiated into adipocytes. Lipid vesicles stained with FABP4 antibody (green), lens magn. 20 ×, (**B**) MSC cells differentiated to chondroblasts stained with Safranin O, lens magn × 5, (**C**) osteoblast-differentiated MSC. Red colour indicates the presence of Ca^2+^ calcium ions (bound by the dye—alizarin red), area 20 × objective, osteoblast.

The isolated cells had the ability to differentiate into three cell lines: adipocytes, chondrocytes and osteoblasts. Cells differentiated to adipocytes were determined by expression of fatty acid binding protein 4 (FABP4) (Fig. 2A). Alizarin red revealed the presence of Ca^2+^ calcium ions in the osteoblast preparation obtained from differentiated MSC (Fig. 2B). MSC differentiated into chondrocytes were stained with Safranin O solution (Fig. 2C).

#### The migration ability of MSC towards cancer cells was confirmed

**Figure 3:**
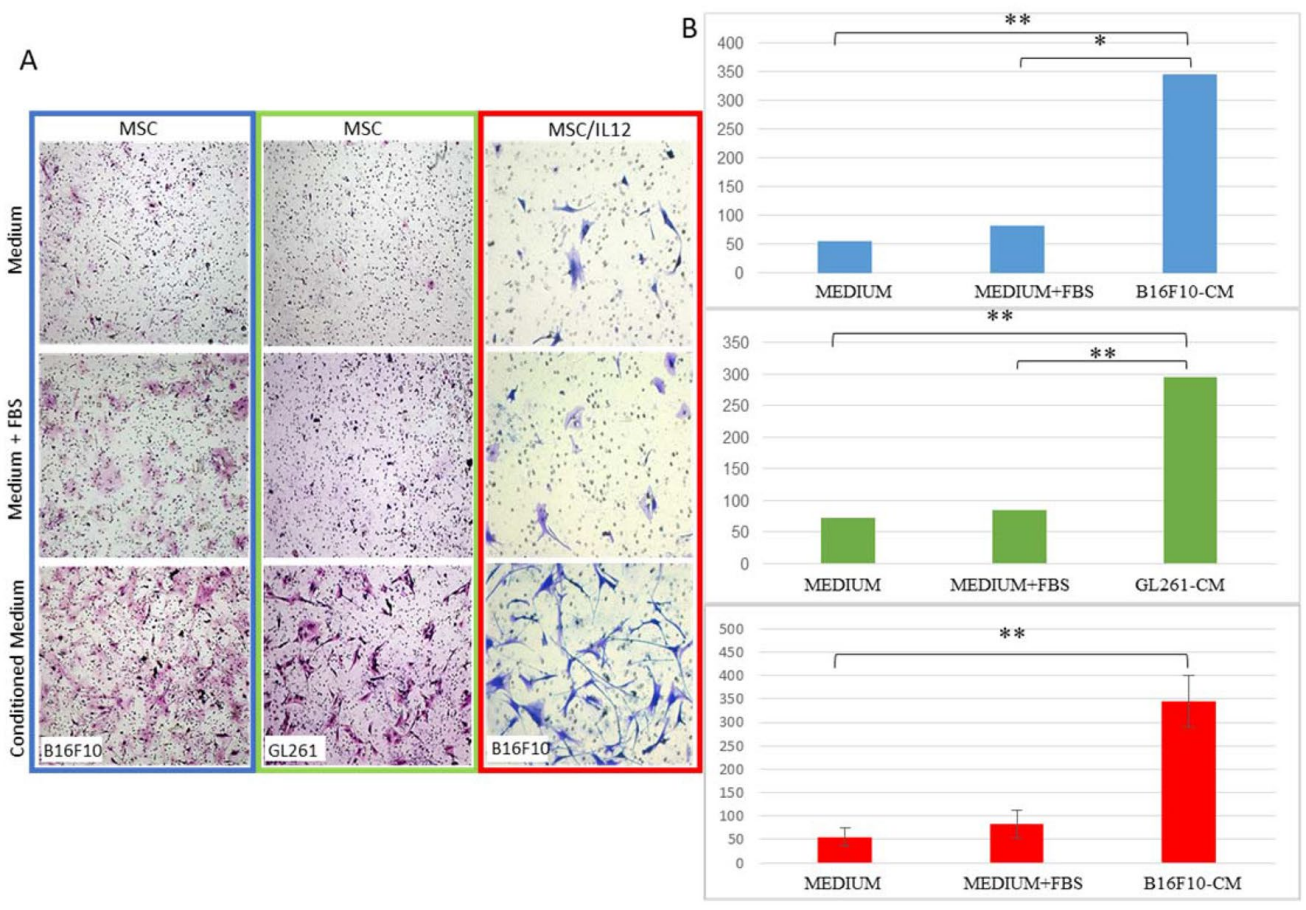
Migration of MSC to chemotactic factors secreted by cancer cells. (**A**) Selected images of MSC and MSC/IL-12 that migrated to control medium, control medium enriched with FBS serum, medium from above B16-F10 cells medium and from above GL261 cells ((lens magn. × 4). (**B**) Sixfold more MSC migrated towards B16-F10 conditioned medium than to control medium, fourfold more MSC migrated towards GL261 conditioned medium than to control medium, sixfold more MSC/IL-12 migrated towards B16-F10 conditioned medium than to control medium. *p < 0.05, **p < 0.005.

**Figure 4:**
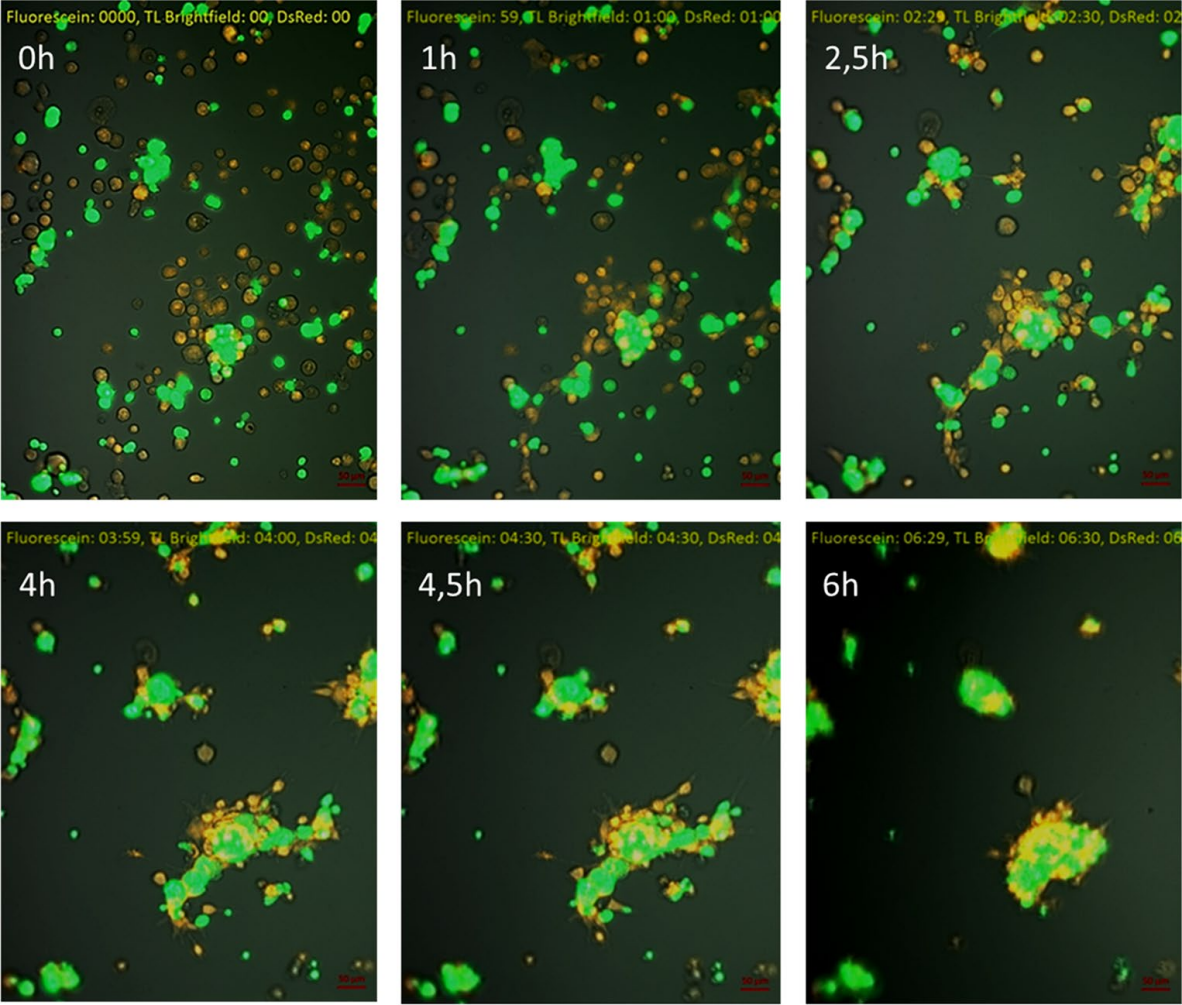
Tropism of MSC (red, PKH) towards GL261eGFP glioma cells (green) in Matrigel. Selected pictures show cells at the beginning of the experiment, after 1 h, after 2.5 h, after 4 h, after 4.5 h, after 6 h. After 2.5 h, tropism of MSC towards glioblastoma cells was observed, after 6 h all MSC visible in the field of view localized near cancer cells (lens magn. × 10).

The migration capacity of MSC and MSC/IL-12 to cancer cells and to media collected from cancer cells (chemotactic agents secreted by the cells) was examined (Figs. 3, 4). The tropism towards the media containing chemotactic factors secreted by cancer cells was examined using Boyden chambers (Fig. 3). MSC migration was confirmed to both media collected from melanoma and glioma cells culture (Fig. 3A). MSC/IL-12 migration to media collected from melanoma cells culture was confirmed (Fig. 3A). The cells migrated through the pores of the sieve towards the tested medium. The number of the cells migrating to the media was counted and compared (Fig. 3B). More than sixfold more MSC as well as MSC/IL-12 migrated to the B16-F10 cell conditioned medium than to the control medium without serum (and fourfold more than to the control medium supplemented with FBS (Fig. 3B). More than 4 times more MSC migrated to the medium from GL261 than to the control medium without serum and 3 times more than to medium with serum (Fig. 3B).

Co-cultures of MSC and GL261eGFP glioma cells were observed to examine the cell mobility (Fig. 4). MSC were stained with PKH26 and showed red fluorescence. The cells were incubated in Matrigel (as a simulation of tissue conditions) under standard culture conditions. The 24-h culture and observations were performed in the microscope chamber for in vivo cell examinations. Over time, there was a gradual accumulation of MSC around cancer cells (Fig. 4). After the first 2.5 h, aggregation of MSC near cancer cells was noticeable (Fig. 4). After 6 h, all MSC visible in the field of view remained clustered around the glioma cells (Fig. 4).

### The cells modification

**Figure 5:**
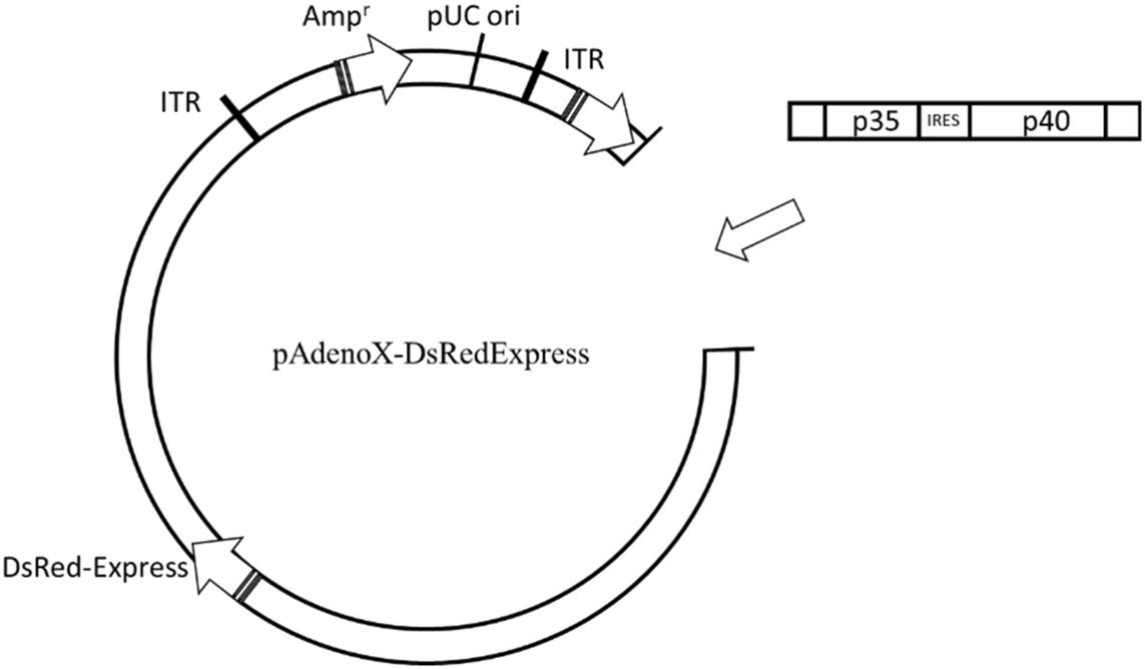
Schematic diagram of the plasmid pAdenoX-DsExpress with the introduced cDNA encoding p35 and p40 IL-12 subunits. The plasmid also contains DsRED marker gene and the gene for ampicillin resistance. The image was drawn using CorelDRAW Graphics Suite X5, 2010.

**Figure 6:**
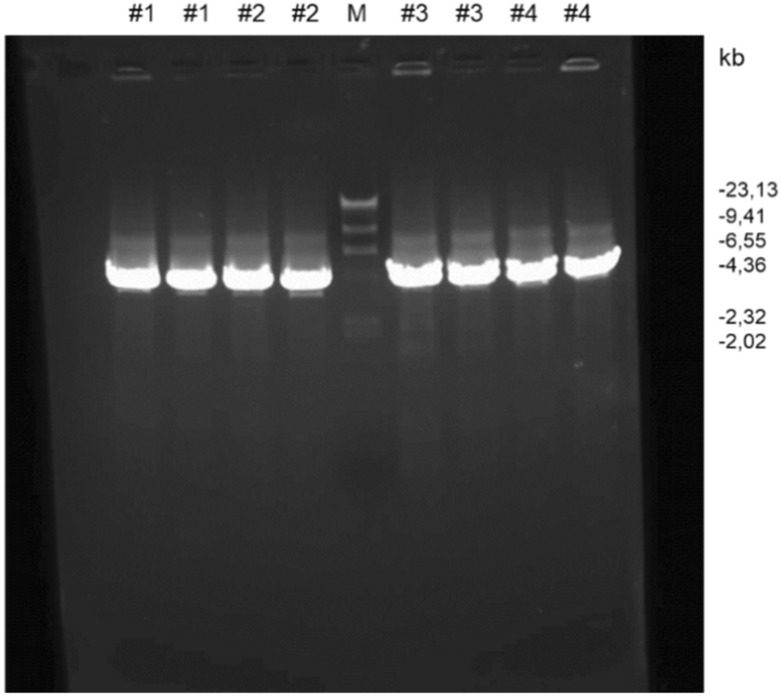
Control of the obtained adenoviral constructs by PCR. 4 selected colonies of bacteria transformed with the vector were subjected to PCR (# 1, # 2, # 3, # 4). Results (in duplicate for each clone) were analysed on an agarose gel. The expected size of positive clones is 4.15 kb. M—DNA size marker Phi X174 HaeIII/Lambda HindIII. All of the clones subjected to the reaction were positive for recombinant DNA content.

The procedure of introducing IL-12 DNA into MSC was successful. First, two subunits of IL-12 cDNA were introduced into pAdenoX-DsRed-Express adenoviral vector (Fig. 5) by cloning. Positive clones were chosen using Colony PCR reaction (Fig. 6) and purified. The sequencing confirmed the cloning efficiency.

**Figure 7:**
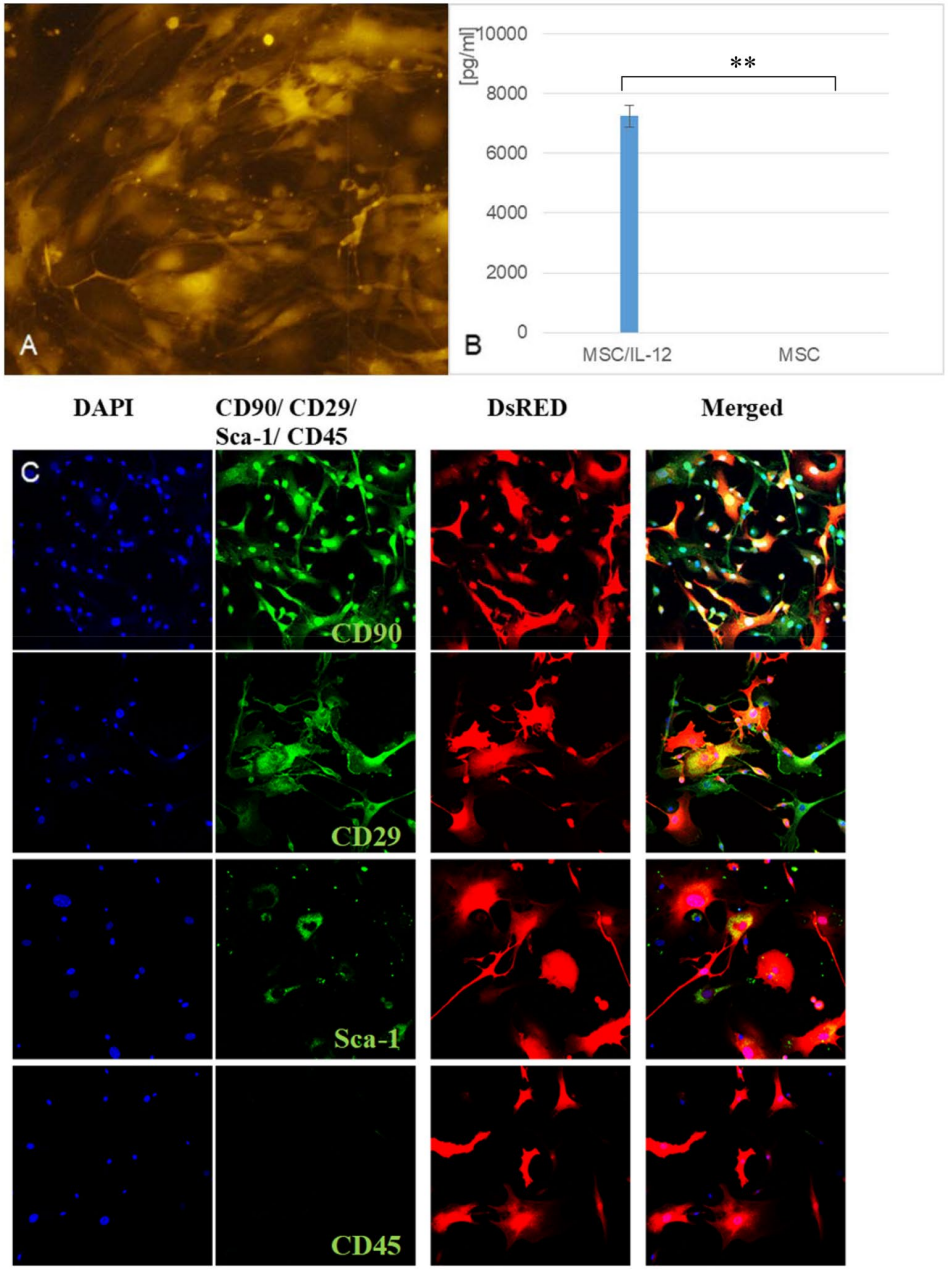
Modified MSC secreted IL-12. (**A**) Transduced MSC expressing dsRED marker gene and red fluorescence, lens magn. × 10 (**B**) Amount of IL-12 protein produced by modified MSC (MSC/IL-12) compared to control MSC. **p < 0.005 (**C**) Expression of selected antigens on the surface of transduced MSC secreting IL-12 and expressing dsRED marker gene, presence of CD90, CD29, Sca-1 and absence of CD45, lens magn. × 20.

A transfection of AdenoX 293 packaging cells using obtained plasmid DNA was conducted and virus isolation and amplification were performed. MSC were transduced with obtained viral particles and incubated until intense red fluorescence was observed (Fig. 7A). The ability of MSC/IL-12 cells to secrete IL-12 was confirmed by ELISA test. Modified cells produced IL-12 in opposite to unmodified cells that do not secrete IL-12 at all (Fig. 7B). The phenotypic characteristic of the transduced cells was determined. Typical markers of MSC: CD90, CD29 and Sca-1 and the absence of the CD45 marker were shown in immunofluorescent staining and microscopic analysis (Fig. 7C).

### Experiments on animals

The modified cells (MSC/IL-12) were used in experiments on animals bearing primary and metastatic B16-F10 melanoma.

#### The single-dose administration of MSC/IL-12 cells significantly reduced the volumes of primary melanoma tumors

**Figure 8:**
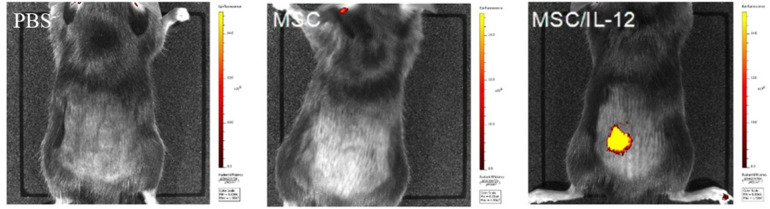
Red fluorescence of modified MSC visualised with IVIS system one day after administered to mice PBS, MSC, MSC/IL-12.

**Figure 9:**
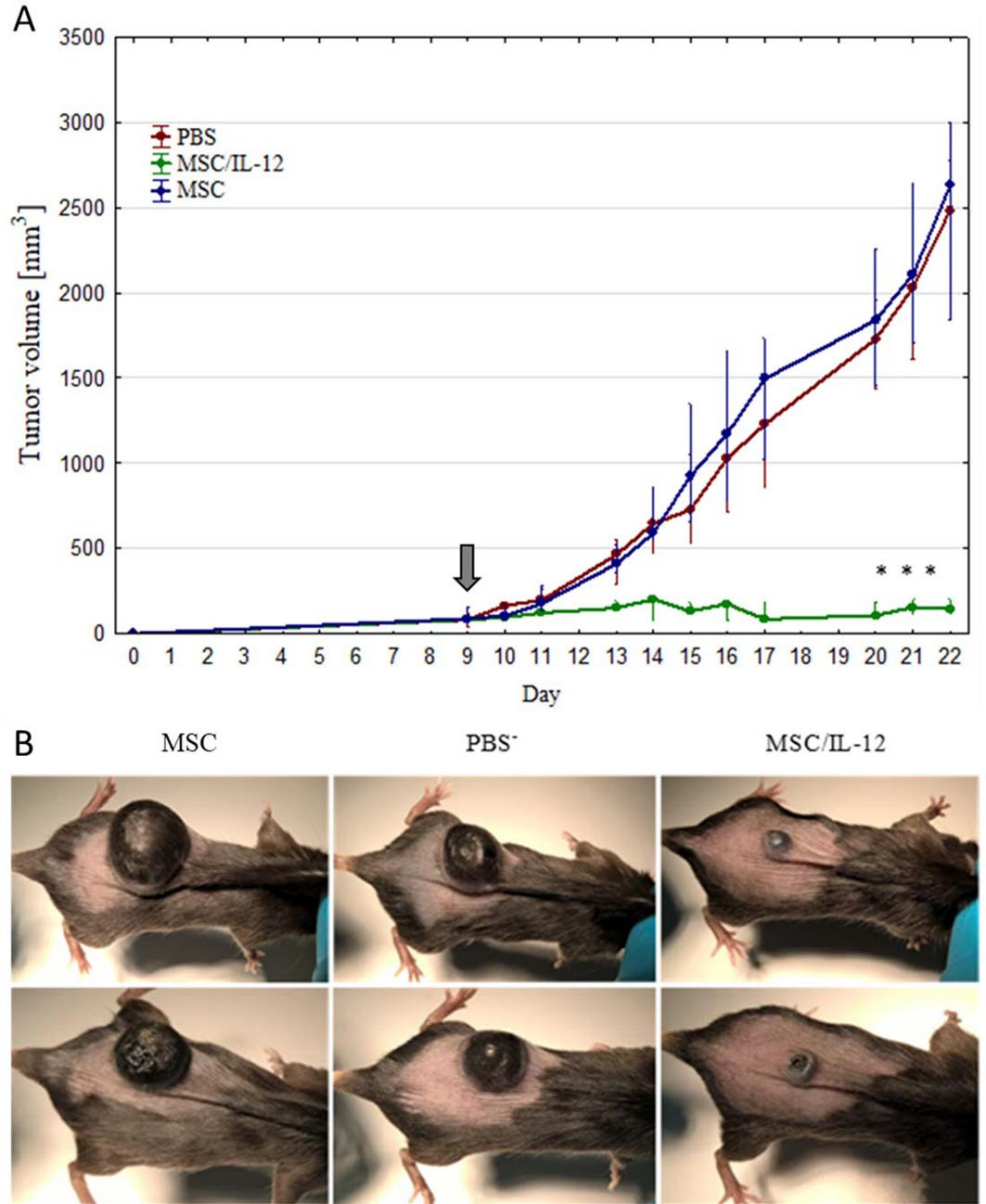
Therapy of mice bearing primary B16-F10 melanoma. MSC/IL-12 inhibited the growth of melanoma tumors. (**A**) 9 days after inoculation with B16-F10 cells MSC, MSC/IL-12 and PBS were administered intratumorally to mice (arrow). On day 20th of the experiment, tumors in mice (n = 5) treated with MSC/IL-12 cells were eightfold smaller comparing to tumors in control (**B**) Representative pictures of animals treated with unmodified MSC, PBS^-^ and MSC/IL-12, * p < 0.05.

IL-12 producing MSC were used in the therapy of mice bearing primary B16-F10 melanoma tumors. 9 days after inoculation of mice with B16-F10 cells MSC/IL-12 were administered intratumorally (Fig. 8). On the 20th day of the experiment, the tumor volumes in mice treated with modified MSC were eightfold lower than in control mice receiving PBS^-^ (Fig. 9A,B). The animals were lively and showed any side effects.

#### A single administration of MSC/IL-12 to the tail vein of animals resulted in a significant reduction in melanoma lung metastases

**Figure 10:**
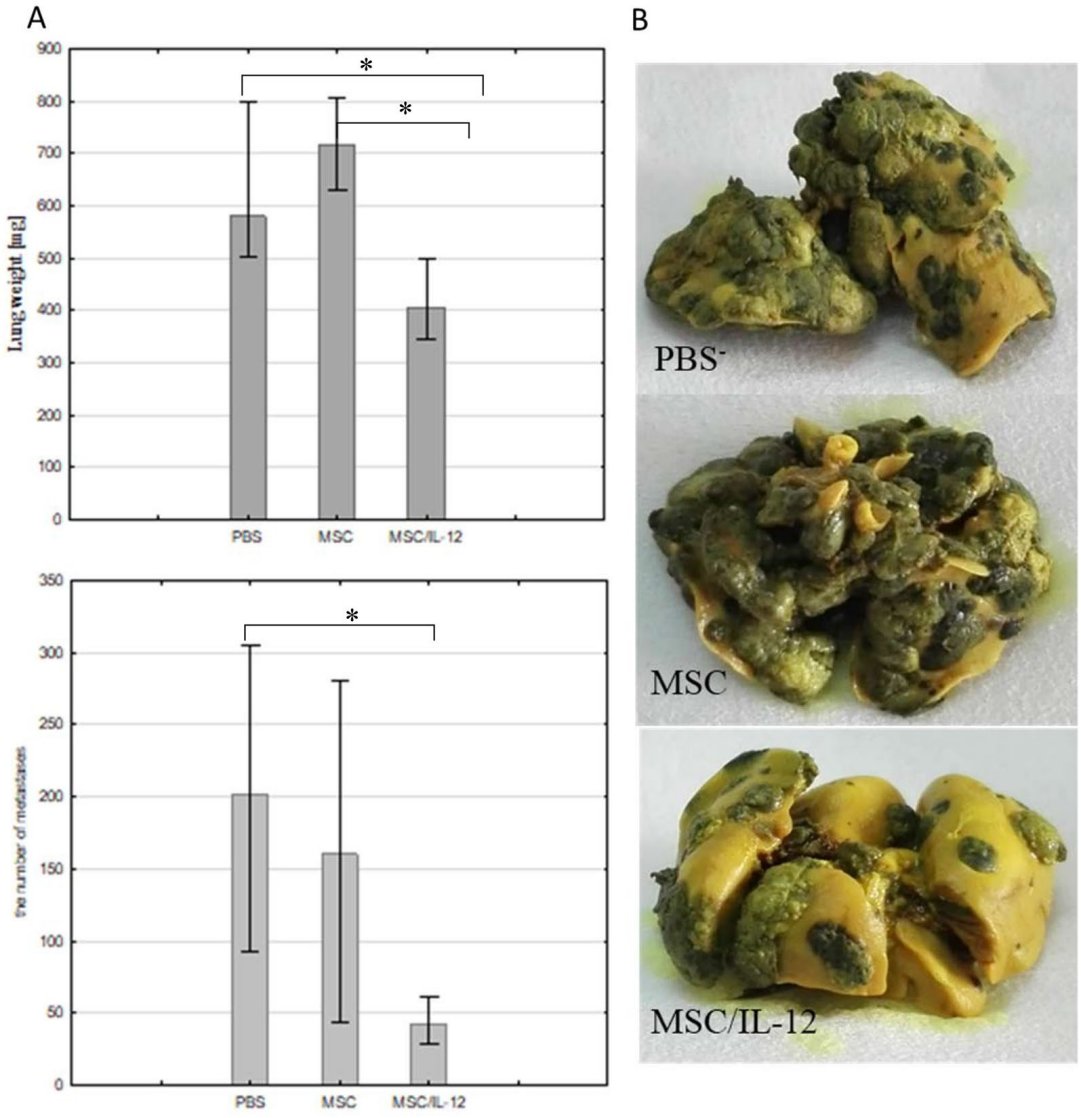
Therapy of mice bearing murine B16-F10 melanoma lung metastases. (**A**) Number and weight of lungs excised from the animals (n = 5). Lungs collected from mice treated with MSC/IL-12 was significantly lighter than lungs from control mice. The number of metastases in the lungs of mice treated with MSC/IL-12 cells was approximately fourfold lower than in mice receiving PBS^−^ (**B**) Representative images of fixed lungs from mice treated with PBS^-^, unmodified MSC, MSC/IL-12, *p < 0.05.

MSC/IL-12 cells were used in the treatment of mice with B16-F10 melanoma lungs metastases. 5 days after B16-F10 cells *iv* administration MSC/IL-12 were given to the tail vein. In 21st day after cells administration the number of lung metastases in mice treated with modified MSC was approximately fourfold lower than in mice receiving PBS^-^ (Fig. 10A). Collected lungs were weighed, and it was noted that the lungs of mice treated with MSC/IL-12 had significantly lower mass than lungs of mice receiving PBS^-^ (Fig. 10A). Metastases in the lungs of treated mice were small, not assembled, did not cause tissue deformation (Fig. 10B). Lung metastases in control mice were large and occupied a significant proportion of lung tissue (Fig. 10B).

### Post-therapeutic analyses

After the experiments on animals, the tumors were excised, collected and the post-therapeutic analyses were performed. To assess the effect of modified MSC on the two most important factors affecting tumor progression: vascularization and immune cells activation, the blood vessel network density and ratio of M1 and M2 macrophages in the tumors of treated and untreated mice were compared.

#### Single, intratumoral administration of MSC/IL-12 cells resulted in a significant reduction in vascular density in melanoma tumors

**Figure 11:**
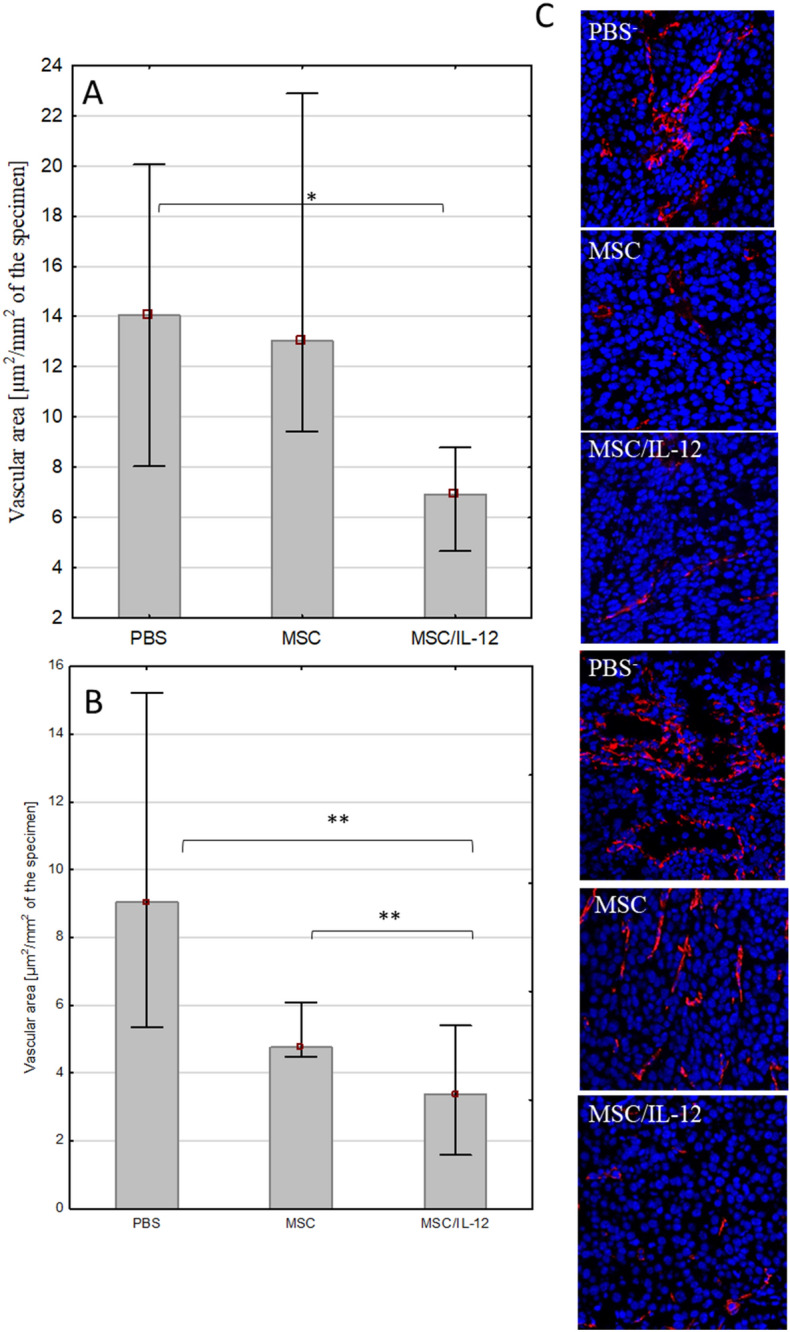
The effect of applied therapy on blood vessels in tumors excised 3 (**A**) and 8 (**B**) days after administration of MSC/IL-12 (n = 5). Area occupied by the vessels [μm^2^/mm^2^ of the preparation]. The area of vessels in tumors collected from mice treated with MSC/IL-12 cells was twofold lower than in tumors from mice that received PBS^-^, both on the 3rd and 8th day of therapy. Representative images of tumor sections from mice treated with PBS, unmodified MSC or MSC/IL-12 (C). Endothelial cells—CD31 (AlexaFluor594, red), cell nuclei (DAPI, blue), lens magn. × 20, *p < 0.05, **p < 0.005.

MSC/IL-12 were administered intratumorally 8 days after the inoculation with B16-F10 cells. Three and eight days after the administration the tumors were collected, fixed and the vascular density was investigated by immunohistochemistry. The vascular surface area (per 1 mm^2^ of the preparation) in group that received MSC/IL-12 was twice lower than in controls both on days three (Fig. 11A) and eight (Fig. 11B) after MSC/IL-12 administration. In tumors collected from treated mice, the vessels were rare and small (Fig. 11C). In control mice tumors, a dense network of mature vessels with significant lumen was observed (Fig. 11C).

#### Single, intratumoral administration of MSC/IL-12 cells resulted in a change in the composition of the macrophage population in tumors

**Figure 12:**
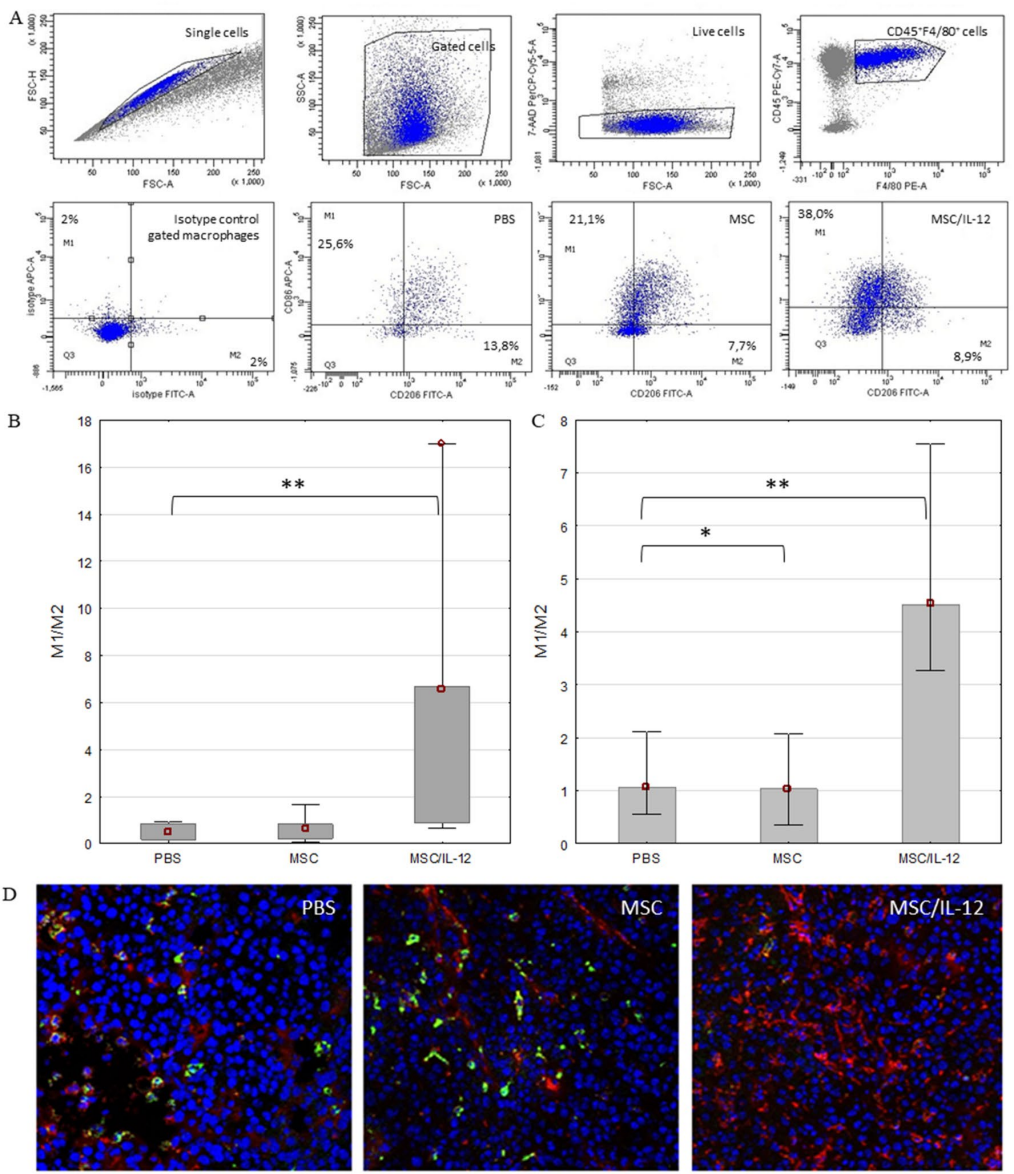
The M1/M2 ratio of macrophages in tumors after the therapy. Flow cytometry gating strategy with representative flow graphs. Macrophages (7-AAD^-^CD45^+^F4/80^+^) from each tumor were gated to appropriate isotype control (**A**). Macrophages isolated from B16-F10 tumors [per 100 mg of tissue, n = 5] collected 8 days after the cells administrations and analysed by flow cytometry. In tumors collected from animals treated with MSC/IL-12 cells, the ratio of M1/M2 macrophages is significantly higher comparing to controls (**B**). Immunohistochemical analyses of frozen sections from tumors (n = 5) collected 8 days after the cells administration. The ratio of M1/M2 macrophages in tumors collected from mice treated with MSC/IL-12 is fourfold higher comparing to controls (**C**). Representative images of tumor sections from mice treated with PBS, unmodified MSC or MSC/IL-12, (F4/80^+^, AlexaFluor594—red, CD206^+^, FITC—green) (D). *p < 0.05, **p < 0.005.

The number of M1 and M2 macrophages in tumors collected from mice treated with MSC/IL-12 compared to controls by flow cytometry. On day 8 after cells administration, mice were sacrificed and tumors were excised for analysis. Tumor cell suspension was stained with antibodies directed against the CD45, F4/80, CD86, CD206, antigens. In tumors collected from mice treated with modified cells, the M1/M2 ratio was 14 times higher than in tumors of control mice (Fig. 12A).

#### The above observations were confirmed by immunohistochemical analyzes of tumor sections

MSC/IL-12 cells were administered intratumorally 8 days after the inoculation with B16-F10 cells. The tumors were collected 8 days after the cells administration; tissue sections were prepared and incubated with antibodies directed against the antigens M1 (F4/80^+^/CD206^-^, AlexaFluor594—red), M2 (F4/80^+^/CD206^+^, AlexaFluor594—red, FITC—green). Macrophages isolated from B16-F10 tumors were collected 8 days after the cells administrations and analysed by flow cytometry (Fig. 12A). The same analysis was performed in the frozen sections (Fig. 7B). The ratio of M1/M2 macrophages in tumors collected from mice treated with MSC/IL-12 was over fourfold higher comparing to controls (Fig. 12B,C). Selected photos of the preparations show the prevalence of M1 macrophages in tumor sections from treated mice. (Fig. 12D).

#### Single, intratumoral administration of MSC/IL-12 cells resulted in an increase of CD8^+^ T lymphocytes

**Figure 13:**
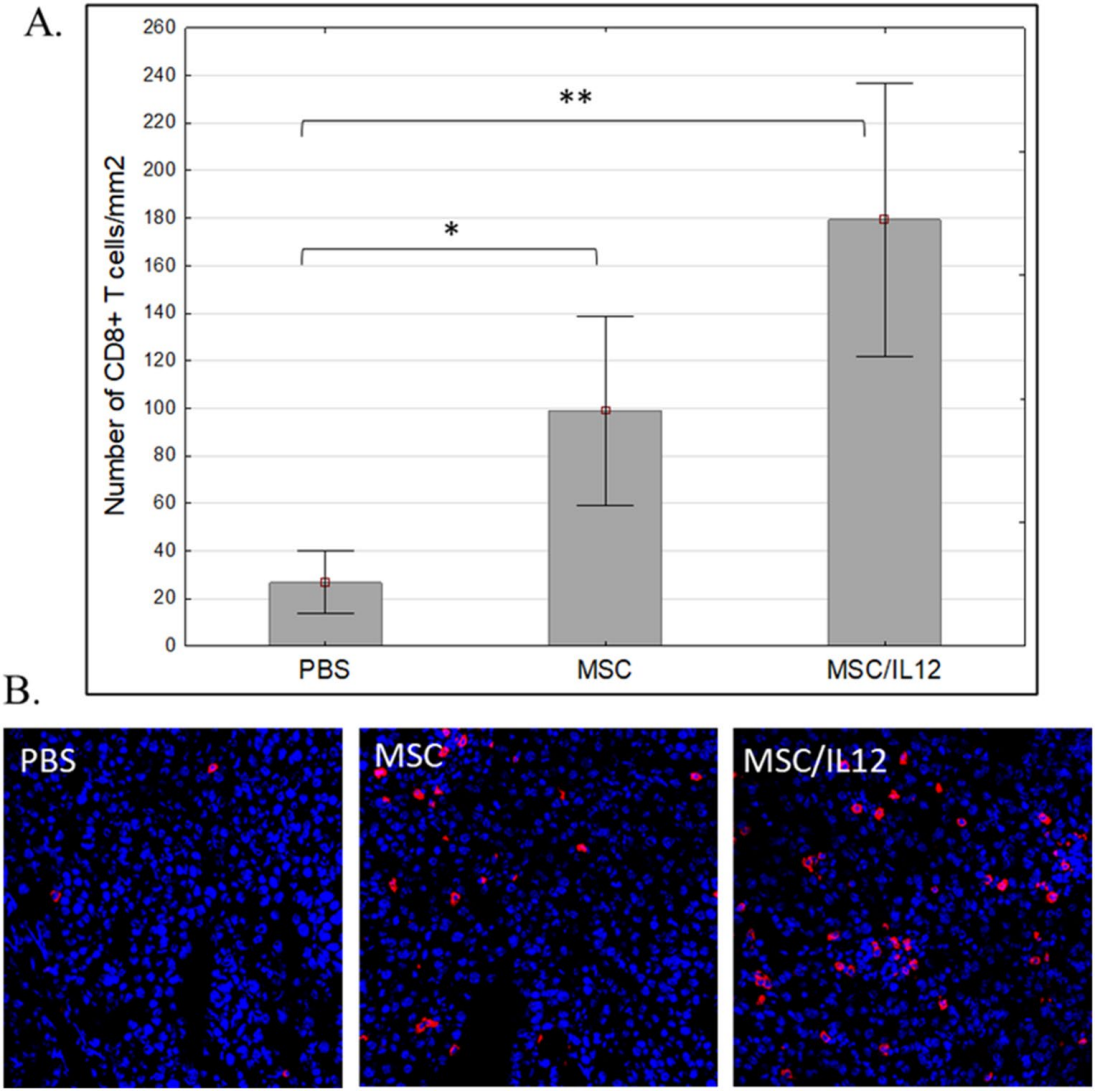
The effect of applied therapy on CD8^+^ lymphocytes in tumors excised 3 days after MSC/IL-12 administration. Total number of CD8 positive T cells was calculated per mm^2^ of tumor section (n = 5). The number of CD8^+^ surface area T cells (per mm^2^ of the preparation) in group that received MSC/IL-12 was over eightfold higher comparing to control and over fivefold higher compared to MSC group (**A**). Representative images of tumor sections from mice treated with PBS, unmodified MSC or MSC/IL-12 (**B**). CD8^+^ T lymphocytes (AlexaFluor594—red), cell nuclei (DAPI—blue), lens magn. 20x, *p < 0.05, **p < 0.005.

MSC/IL-12 cells were administered intratumorally 8 days after the inoculation with B16-F10. Three days after the administration the tumors were collected, fixed and the number of CD8^+^ T cell was determined by immunohistochemistry (Fig. 13B). The number of CD8^+^ T cells (per 1 mm^2^ of the preparation) in group that received MSC/IL-12 was over eightfold higher comparing to control and over 1.5-fold higher compared to MSC group (Fig. 13A).

## Discussion

In the last over a dozen years IL-12 has become the subject of interest of researchers as one of the most effective antitumor cytokine. IL-12 as a mediator of inflammation interacts with a number of cells of the immune system, acting as a bond between the adaptive and innate immune responses. IL-12 as a potent immunostimulant activates T lymphocytes and NK cells as well as triggers release of IFN-γ, all of which induce a strong immune response directed against cancer cells^22,23^. This effect is considered responsible for stimulating IL-12 related production of IFN-γ. IFN-γ also reduces the ability of tumor cells to produce VEGF^24^.

Our group is exploring the IL-12 based therapeutical approach for almost 20 years. Anti-tumor effects of IL-12 have been proved in models of renal cancer^25^ and in anti-melanoma combination therapies with cyclophosphamide^26^, endoglin-based DNA vaccine^27^, CAMEL peptide^28^, _D_-K_6_L_9_ peptide^29^, anti-vascular ABRaA-VEGF121 chimeric protein^30^ and tumor cell lysate^31^. Similar studies have been carried out in many other laboratories, where the efficacy of therapy using adenoviral plasmid with IL-12 in liver, colon and pancreatic cancer models was tested^32^.

All the above therapeutic solutions have proved effective in inhibiting the growth of tumors and showed satisfactory effects only after administration of IL-12 directly in the vicinity of the tumor. However, there are serious limitations to use IL-12 in therapy. Long-term systemic administration of high doses of IL-12 caused side effects like fever, fatigue, hematological disorders, hyperglycemia, liver damage, and acute colitis. There have been deaths among patients undergoing clinical trials with its use^33–35^. There is still a need to find a way to administer the cytokine intravenously without overall toxicity. It would be necessary to treat the patients bearing with hard-to-reach tumors.

The aim of the work was to develop a system in which cytokine may be administered intravenously without toxic side effects. In this study, mesenchymal stromal cells were used as carriers of IL-12 cytokine.

MSC are immunologically privileged, do not induce an immune response in the recipient after transplantation^9,36–39^ and show strong tropism to tumors sites^40–43^. Inflammation and hypoxia are attractors to MSC, causing their migration into the damage zone, in which a number of chemokines, adhesion molecules and metalloproteinases participate e.g. EGF, VEGF, PDGF, IL-8, IL-6, FGF, SDF-1, G-CSF, MCP-1, HGF, TGF-β and uPA^44–47^. Barcellos-de-Souza et al. demonstated that only MSC have a tropism to tumors. Such tropism was not observed for normal fibroblast^48^. Similar results were presented by Liu et al.^49^.

In our study, we confirmed the ability of MSC to migrate towards the media collected from cancer cells (Fig. 3) More than sixfold more MSC migrated to the B16-F10 cell conditioned medium than to the control medium. More than 4 times more MSC migrated to the medium from GL261 than to the control medium. MSC migration was also observed towards cancer cells themselves (Fig. 4). The tropism was examined on Matrigel in co-culture of MSC with GL261 glioma cells. After the first 2 h of incubation, aggregation of MSC near cancer cells was noticeable. After 6 h, all MSC visible in the field of view remained clustered around the glioma cells. Owing to these specific migratory abilities MSC are an excellent vehicle for transferring therapeutic factors.

The employment of mesenchymal stromal cells for the transfer of anti-cancer agent as IL-12 appears to be a promising therapeutic strategy against melanoma. The use of genetically modified IL-12 producing cells has many benefits. Transfection or transduction may be optimized ex vivo without exposing the recipient organism to high, toxic doses of the cytokine. It is possible to use as gene carriers the cells showing natural tropism to tumors^50,51^. In our opinion, the most important is that IL-12-modified MSC trigger the cascade reaction activated by IL-12 protein. Previously, using the adenovirus vectors, the IL-12 gene was introduced into fibroblasts^52^, dendritic cells in therapies against leukemia^53^ and melanoma^54^ and T cells in therapy against the thymoma^55^. Mesenchymal stromal cells seem to be an effective carrier of the IL-12 gene. MSC secreting IL-12 were used in the therapy of mice bearing glioblastoma. Intracranial administration inhibited the growth of tumors and increased the survival of animals^51^. IL-12 secreting MSC were used in the treatment of mice with Ewing sarcoma, where tumor growth inhibition was also observed^56^.

In our study MSC secreting IL-12 (MSC/IL-12) administered directly to the B16-F10 melanoma tumors significantly inhibited their growth. Ten days after the administration of the cells, the volumes of tumors in mice that received MSC/IL-12 cells were eightfold lower than in mice treated with PBS^-^ (Fig. 9).

In the study MSC/IL-12 cells were administered to the tail vein of mice 4–5 days after experimental B16-F10 metastases initiation. The lungs were collected and analyzed after 21 days from administration of the modified MSC. In the lungs of mice treated with MSC/IL-12, fourfold less metastases were formed than in the lungs of control mice (Fig. 10). MSC are larger than hematopoietic cells, so as many as 80% of them are retained in the pulmonary capillaries a few minutes after *iv* administration^57^. At the basis of communication between MSC and endothelial cells are adhesive interactions between molecules on the surface of MSC and endothelial cell receptors. The adhesion of MSC to the pulmonary vascular walls is mainly due to the VCAM-1 adhesion-protein ligand^58^. After administration of the MSC to the bloodstream of the animal, the cells are located in the pulmonary alveoli. Due to the presence of adhesins and integrins (CD29, CD44) on the surface of the MSC membrane (Fig. 1) they adhere to the walls of the blood vessels. MSC are located in the vicinity of macrophages residing in the lungs and in the vicinity of tumor cells^58^. Studies indicate that MSC remain in the lungs for up to 4 days after administration^57^. Bortolotti et al. and Braid et al. indicated that the retention time depends on route of administration and source of MSC^59,60^. Both of these work indicate that bone marrow derived MSC are present in the place of injection for 1–2 days after cells injection and afterwards their number decreases significantly and quickly. On days 14- 21, only single MSC are visible at the injection site^59,60^. Doucette et al. also showed that MSC have tropism to glioma and that the retention of these cells lasted until the 10th day when the MSC were undetectable^61^. Nystedt J et al. in their work showed that bone marrow derived MSC have slower lung clearance than umbilical cord blood MSC^62^.

IL-12 works comprehensively, modifies the microenvironment of tumor. Single, intratumoral administration of MSC/IL-12 cells, 8 days after the inoculation with B16-F10 resulted in a significant reduction in vascular density in melanoma tumors. Three and eight days after the cells administration the vascular density in group that received MSC/IL-12 was over twice lower than in controls (Fig. 11).

Tumor blood vessels are abnormally tortuous—their chaotic architecture greatly affects fluctuating slow blood flow and exacerbate metabolic mismatch between supply and demand what leads to progressive hypoxia. IL-12 modifies the tumor microenvironment: reduces the number of blood vessels. The anti-angiogenic efficacy of IL-12 is probably due to the stimulation of IFN-γ production^24,63^. IFN-γ induced by IL-12 reduces the secretion of VEGF by tumor cells. Therapy with the use of IL-12 lowers the production of metalloproteinases, important in tissue remodeling during neoangiogenesis. IL-12-stimulated production of IFN-γ also contributes to inhibiting the activation of α_V_β_3_ integrins and downregulating the expression of ICAM-1 and VCAM-1 adhesion proteins on the surface of endothelial cells. IL-12 stimulates the production of cytokines and anti-angiogenic chemokines^64–66^. NK cells that accumulate around the blood vessels in the presence of IL-12 have cytolytic properties toward endothelial cells^67^.

IL-12 modifies the tumor microenvironment—changes the proportion of the macrophage populations. Macrophages are the most plastic population of cells in the immune system. They undergo specific activation and occur in two functionally different phenotypes presented in response to environmental conditions^68^. These are the classically activated macrophages M1 (proinflammatory) and alternatively activated M2 macrophages (immunosuppressive). The specific plasticity allows them to functional reprogramming depending on the stimulus available^69^. M1 macrophages are associated with the initiation and maintenance of inflammatory response, M2 with inflammation quenching and tissue regeneration^70,71^. Tumor-Associated Macrophages (TAM) phenotype resembles the M2 macrophage phenotype. TAM (M2) produce tumor growth and invasive factors such as growth factors (EGF and VEGF), cytokines, enzymes supporting the process of angiogenesis (MMP9), inhibit the expression of anti-cancer factors such as IL-12 and accumulate at hypoxic sites^72,73^. TAMs show strong immunosuppressive properties through the production of anti-inflammatory cytokines and chemokines^73,74^.

In our study MSC/IL-12 cells elicit changes in the proportion of macrophage populations in tumors. A 14-fold increase in the ratio of M1 to M2 macrophages was observed in tumors after MSC/IL-12 treatment comparing to control (Fig. 12). These cells are probably co-responsible for therapeutic effect stimulated after MSC/IL-12 cells injection.

Our results showed that the administration of IL-12 stimulates infiltration of CD8^+^ T lymphocytes in tumors after MSC/IL-12 administration (Fig. 13), which is also confirmed by others^24,75–77^.

IL-12 stimulate also the production of IFN-γ by T cells and NK cells and induce a strong anti-tumor response^78^. INF-γ activates M1 macrophages^73^, causes the repolarization of immunosuppressive M2 macrophages (TAM) to proinflammatory M1 macrophages^78–81^.

## Summary

The effectiveness of the proposed system, i.e. the inhibition of melanoma progression, results from the use of both: the characteristics of the carrier and the drug transported. MSC show tropism to cancer cells and release the therapeutic protein in their vicinity. IL-12 has anti-angiogenic and immunostimulatory activity and leads to inhibition of tumor progression.

Modified mesenchymal stromal cells secrete IL-12 that inhibit the progression of murine B16-F10 melanoma. The proposed therapeutic system inhibits the growth of primary tumors and metastases in the lungs. The probable mechanism responsible for the effectiveness of the therapy is the inhibition of angiogenesis evoked by MSC/IL-12 cells and the increase in the percentage of M1 macrophages and CD8 T lymphocytes in the tumor tissue.

## Supplementary Information

Supplementary Information.
