# Supplementary material for: Mesenchymal stromal cells as carriers of IL-12 reduce primary and metastatic tumors of murine melanoma
Source: Sci Rep. 2021 Sep 15;11:18335. doi: 10.1038/s41598-021-97435-9 (PMC8443548; doi:10.1038/s41598-021-97435-9)
Supplement: Supplementary file 1 — Supplementary Information. [file 41598_2021_97435_MOESM1_ESM.docx]

Mesenchymal stromal cells as carriers of IL-12 reduce primary and metastatic tumors of murine melanoma

**Natalia Kułach^1^, Tomasz Cichoń^1^, Ewelina Pilny^1^, Magdalena Jarosz-Biej^1^, Marek Rusin^1^, Justyna Czapla^1^, Alina Drzyzga^1^, Sybilla Matuszczak^1^, Stanisław Szala^1^ and *Ryszard Smolarczyk^1^**

^1^Center for Translational Research and Molecular Biology of Cancer, Maria Skłodowska-Curie National Research Institute of Oncology, Gliwice Branch, Wybrzeże Armii Krajowej Street 15, 44-102 Gliwice, Poland

***** Correspondence: Ryszard.Smolarczyk@io.gliwice.pl; Tel.: +48 32 278 97 51


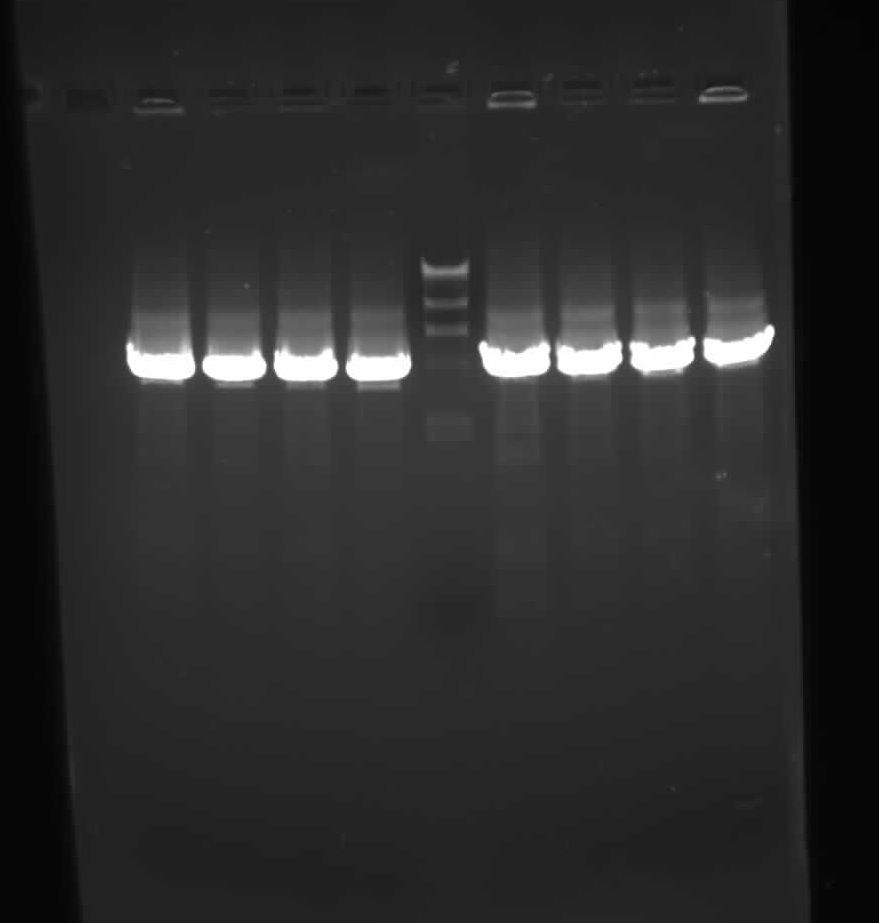


Fig.1. Original photo of the gel from Fig. 6 in the main text containing obtained adenoviral constructs subjected to control PCR reaction.
